# Implementation process and challenges for the community-based integrated care system in Japan

**DOI:** 10.5334/ijic.988

**Published:** 2014-01-20

**Authors:** Takako Tsutsui

**Affiliations:** National Institute of Public Health, Ministry of Health, Labour and Welfare, Saitama, Japan

**Keywords:** Long-term Care Insurance System in Japan, community-based care, integrated care

## Abstract

**Background:**

Since 10 years ago, Japan has been creating a long-term vision to face its peak in the number of older people that will be reached in 2025 when baby boomers will turn 75 years of age. In 2003, the government set up a study group called “Caring for older people in 2015” which led to a first reform of the Long-Term Care Insurance System in 2006. This study group was the first to suggest the creation of a community-based integrated care system.

**Reforms:**

Three measures were taken in 2006: ‘Building an active ageing society: implementation of preventive care services’, ‘Improve sustainability: revision of the remuneration of facilities providing care’ and ‘Integration: establishment of a new service system’. These reforms are at the core of the community-based integrated care system.

**Discussion:**

The socialization of long-term care that came along with the ageing of the population, and the second shift in Japan towards an increased reliance on the community can provide useful information for other ageing societies. As a super ageing society, the attempts from Japan to develop a rather unique system based on the widely spread concept of integrated care should also become an increasing focus of attention.

## Introduction

Explaining why the community-based integrated care system was implemented in Japan cannot be done without addressing the issues related to the ageing of the population and the decline in birthrates. In 1950, the proportion of older people in the population was under 5%, but already exceeded 7% in 1970 (ageing society) and 14% in 1994 (aged society). Recently, as the total population started to decline while the older population kept increasing, it is expected that the rate of older people will reach 40.5% in 2055, which means that more than one third of the population will be over 65 years of age [1].

The ageing of the population, as a structural transformation that occurs in every age group of the population, has a major influence not only on the medium-term economic growth but also on business administration, industry and market through changes in the employment and consumption structure. Moreover, it reinforces the political influence of the elderly population, which may have an impact on the social security system, such as the health care and pension system.

The social security reform bill enacted in Japan on 21 August 2013 addressed a number of issues, including: the division and collaboration of clinical functions via a formulation of the community-based medical care delivery system; a revision of the medical care cost burden shared by patients aged 70–74; new measures to handle intractable diseases; and a reduction of the pension eligibility period from 25 to 10 years. In every country, the ageing of the population implies drastic transformations at the political, economic and societal level.

The ageing of the population and the decline in birthrates has been particularly brutal in Japan and became a major obstacle to the establishment of a sustainable social security system. Those changes became a notable issue especially for the health care system, which was modified by a revision of the Medical Insurance Act in June 2006 (effectively implemented after 2008). Even according to the most likely scenario of the government on demographic estimations, the ageing of the population and the decline in birthrates will keep progressing for another 50 years from now. The rate of older people is 24.2% in 2012 but, as mentioned earlier, it should reach 40.5% in 2055 and then remain at this level for a while.

Health expenditures for older people over 65 are 4.3 times higher than for other age groups and the majority of costs are covered by the working-class through the payment of taxes and medical insurance premiums. This heavy burden born by the working-class has and will have an important impact on the Japanese economy.

It has to be kept in mind that demographic ageing occurs at a different pace in different geographic regions of the world, and that within each single country this evolution is quite different in urban and rural areas.

In Japan, as a result of the baby boom, the population of older people over 75 years of age will reach its peak in 2025.

In preparation for this situation, the government has set up in 2003 a study group called “Caring for older people in 2015” which suggested for the first time the creation of a community-based integrated care system.

The Social Security National Committee also defined the way medical, long-term and social care should be provided through the “Vision of hope and relief for long-term care” [2] and created the Fifth Plan for the Long-Term Care Insurance starting in April 2012. This plan was created to allow a smooth management of insurance benefits by local governments. More precisely, it includes two different plans: one made for local municipalities and the other made for prefectures and city governments. In accordance with the Long-term Care Act, the plans, which determine the amount of insurance premium, are revised every three years.

The government also formed a Research Committee on community-based integrated care in 2008 to examine and resolve issues associated with the creation of a care system where medical, long-term and social care are provided altogether in a given community (i.e. community-based integrated care). This committee summarized in May 2009 some recommendations in a paper addressed to the government [3]. The paper argued for the need to clarify the various types and roles of care homes and services covered by the Long-Term Care Insurance and to build a system providing care services at home. The role of community-based integrated care centres, which have been implemented in every district (delimited by a school area, covering around 20,000 inhabitants) since 2006, is also expected to keep growing in importance. However, some points of controversy still remain regarding the allocation of financial resources. For example, no decision has yet been taken regarding the eventual need to increase public expenses for the Long-Term Care Insurance System.

This paper will present the main issues addressed in this report, explain its context and discuss the political measures required to implement the community-based integrated care system of Japan, that is, a system which ‘enables citizens to keep living in a familiar environment, regardless of the type of housing, through the use of various services provided locally, around the clock and 365 days a year’.

This paper will review the background to the implementation of this system and provide an overview of political reforms regarding Long-Term Care Insurance System based on the concepts of community and integrated care.

## Definition of the community-based integrated care system in Japan and reasons behind its creation

The community-based integrated care system of Japan is defined in the report from the Research Committee on community-based integrated care as: ‘a system in the community which provides appropriate living arrangements and appropriate social care such as daily life support services in addition to long-term and medical care to ensure health, safety and peace of mind in everyday life’. The ideal size of each community is defined as an approximate range of 30-minute walk, which represents a school district in Japan. The report from the National Assembly on Social Security also stated that it was necessary to build a system to provide social care such as daily life support services in addition to long-term and medical care in a comprehensive and seamless manner within a community [4].

The Research Committee on community-based integrated care also pointed out the three main following topics that needed to be dealt with.

The first topic concerns the provision of medical care at home. Most Japanese currently die in hospitals or health care facilities (Table 1). The rate of persons dying at home does not exceed 13%, which is very low compared to other countries. This is one major reason why the committee suggested the implementation of a community-based care system that integrates health and long-term care.

However, there is still a lack of awareness on the part of municipalities regarding the need to integrate health and long-term care services, even though they have the responsibility to actively promote interactions with the medical field (both primary and secondary care) inside each community. Municipalities are called insurers in Japan, as they are responsible for implementing the Long-Term Care Plan and for determining insurance premiums by looking at the balance between the needs of the population and the quantity of services provided in the area. In Long-Term Care Insurance System, prefectures support the municipalities, while the national government decides the overall direction of the system.

The second topic is the support of the system providing long-term care services and the increase of residential care homes. Currently, the number of insured people (4,550,000 beneficiaries) in Long-Term Care Insurance System is two times higher than it was when the system was implemented in 2000 (2,180,000 beneficiaries) [5–7]. As a result, a three-year plan beginning in 2009 was made to ensure the establishment of 160,000 new places in various types of residential care homes and to recruit 400,000 additional professionals to provide home care services. In reality, the plan was even extended one more year, as only 140,000 new places were established after three years.

In Japan, 85% of older people own their house but when growing older, it is not uncommon that they have to move out and get closer to the city because it becomes difficult or inconvenient to live in their home. However, moving out might be an option for large- or moderate-income families but not for people with low incomes. For this group of people, there is currently a lack of appropriate housing opportunities.

The community-based integrated care system requires the existence of home settings where people, regardless of their income, can live long and safely.

However, especially in the big cities, the implementation of public housing facilities is still insufficient considering the expected increase in the elderly population. This issue is very difficult to resolve due to the lack of detailed assessment of the existing housing and the development of a business exploiting abusively the social security benefits given in cash to low incomers.

The committee concluded that insurers (municipalities) should have the responsibility to find a way to meet the high needs for care services and to determine how many professionals should be recruited to deal with this increase in insured persons.

The committee also stated that each local government should specify their own strategy to build a system of integrated support to secure the implementation of the Long-term Care Plan, and other plans concerning medical services and housing. The nature of these plans may differ greatly depending on the degree to which integrated support already exists in the community.

Moreover, insurers should establish this strategy in cooperation with community-based integrated care centres, informal sector and non-profit organisations, especially in the case of communities with a low density of population, because it may vary depending on the local needs in human resources.

The third topic concerns the plan for preventive care and daily life support services, which both involve the inhabitants of the community as volunteers working under the supervision of health nurses working in community-based integrated care centres and health care centres. These community activities should be promoted as they lie at the foundations of the community-based integrated care system.

On this topic, the community support project and the community-based integrated care centre support a programme, which stipulates that some directives for the community-based integrated care centres were established. The plan for preventive care is a part of the plan for community support and is financed by the government subsidies (Table 2). This plan is independent from the preventive care benefits provided to elderly requiring support as part of Long-Term Care Insurance System [8].

Community-based integrated care centres play a major role in community care. They include health nurses, social workers and care managers, all working as a team. Using funds from the Long-Term Care Insurance System and from taxes, these centres are increasingly being established by municipalities since 2006 and should ultimately exist in every district (delimited by a school area, which covers around 20,000 habitants). The centres have three roles: first, the implementation of various preventative care services (e.g. preventative benefit care management, started in 2003, and the preventative care project, established in 2006); second, outreach and counselling for elderly in need of care, through the use of various community networks; and third, continuous and comprehensive care management support that includes supervision of care managers.

Concerning the second role mentioned above, it seems that in municipalities with rich social resources, it may be possible to leave the matter of rebuilding networks of informal caregivers (neighbours, etc.) to specialists without interfering but, in other municipalities, it is necessary to build a framework to organise networks and to implement organisations in charge of coordinating community activities and social welfare councils. Those councils originated from private charitable organisations supported by the state during the war and the prewar period. They are now private bodies established in each local government. Their current goal is to promote social welfare and support informal caregivers. The collaboration between these councils and the community-based integrated care centres is a key challenge of the system.

Regarding this third topic, small-scale multifunction residential care facilities were also implemented and various community services were designed, including out-of-hours home care services. These services include 24-hour routine home-visit services and need-based care services. In 2006, home-visit services provided at night were also introduced but the lack of providers and the lack of care management procedures to capture the need of users hindered the use of such services. From this moment, local governments selected providers in charge of covering a specific geographical area and care teams of nurses and care staff started to be in charge of the planning of services.

In the community-based integrated care system, the focus is no longer on care by society, which used to be the mainstream concept since the implementation of a social insurance system, but on care by community (Table 3) [9]. This suggests a reactivation of the mutual help that may still exist within families or among residents of a same community.

Before the implementation of the Long-Term Care Insurance System, the great majority of elderly persons were taken care of and supported financially by their family (mostly the elder son's family). Concretely speaking, the spouse of the elder son (the daughter-in-law) of the family was expected to become a caregiver. It was also commonly accepted that sons, especially the elder son and his family, are in charge of the funerals, even when they live in urban areas.

The implementation of the Long-Term Care Insurance System can be seen as an attempt to substitute care provided by the family for social services. However, Long-Term Care Insurance System provides in-kind benefits to those requiring long-term care, but does not provide cash benefits to family caregivers, even though such a system was once considered.

Four main reasons were given to justify this decision.

Firstly, there was a risk that these allowances would reinforce the position of daughters-in-law as family caregivers. This was hard to accept as the Long-Term Care Insurance System just removed part of the care burden from their shoulders.

Secondly, it is difficult to guarantee that cash benefits would be used for care delivery purposes. Thirdly, these benefits were already forbidden in the Medical Care Insurance, as the risk of fraud is higher when cash is given to relatives. Finally, the risk of maltreatment from family caregivers may have been increased.

As for the impact of the implementation of Long-Term Care Insurance System, it appears that the number of care providers increased and that the number of daughters-in-law providing care decreased. Obviously, some other factors such as social transformations may have played a part in this trend. Even after the implementation, the proportion of spouses, sons and daughters as caregivers remained high. Family care is still at the core of the current care delivery system in Japan.

The concept of community-based integrated care in Japan does not define clearly the position of family caregivers. However, considering the decrease in multi-generational households, one challenge and goal of the system is to enable the delivery of care 24 hours a day and all year long.

## International trends towards integrated care and the Japanese community-based integrated care system

According to previous international studies, the goals of integrated care are to improve access to care, the quality of care and the sustainability of the care system [10].

These goals are crucial considering the recent increase in patients with chronic conditions who need a long-term universal and continuous coverage more than acute medical care. A similar situation can be seen in Japan, and it naturally led policymakers and specialists of the health care system to follow a similar path, which is to reexamine the structure of the long-term care and health care systems to improve coordination and integration. This explains the reform of Long-Term Care Insurance System in 2006 towards a community-based care, the search for a new definition of community-based integrated care in Japan in 2008 and 2009, and the revision of the remuneration system for medical care and long-term care services in 2012.

Although the policymakers behind these reforms were not directly influenced by international models of integrated care, Denmark's 24-hour home care system may have had an influence on the 24-hour services established in Japan in 2012 and on the establishment of group homes providing both long-term and medical care. This type of system was already implemented in Denmark, where drastic reforms were made since the 1990s to stop the construction of residential institutions. Japan, 20 years later, also made some attempts to integrate residential care and home care providers, but a strong opposition from the associations of various types of long-term care facilities started to rise in Japan immediately after this idea was suggested.

Some long-term care facilities may have felt threatened by the concept of community-based integrated care as it promotes home care services and could lead to a decrease in the use of facilities. In order to handle this opposition, it is important to continue to promote research studies related to integrated care that aims to dissolve the divide between institutional and home care.

The community-based integrated care system is a framework adapted to the characteristics of the community and which utilizes the resources of each community.

Agents of the community-based integrated care system include users (elderly persons), caregivers (family or else), residents of the community, municipalities, prefectures and city governments, the state, long-term care providers, private businesses, Non-Profit Organisations, community associations. This system has many layers such as the field level, the community management level and the coordination level between various professionals. Policymakers in Japan decided that an entity assuming the leading role should be established at each of these levels. This approach is influenced by previous research [11,12], showing that insufficient integration in any one of the three levels (system, organizational and clinical integration) may hinder the whole integration process.

According to Lloyd and Wait [11], one of the most important points in the integrated care approach is that it secures outcomes and ensures the continuity as well as the quality of care. According to Kodner and Kyriacou [12], integrated care is defined as ‘a set of techniques and organizational models designed to create connectivity, alignment and collaboration within and between the cure and care sectors at the funding, administrative and/or provider levels’.

However, the definition of integrated care and the extent of integration may vary considerably according to national specificities, the type of funding system, cultural traditions and welfare pathways. This suggests that, even though many countries are facing similar challenges in the context of an ageing population with increasing long-term care needs, the situation in each country regarding health care systems and political measures is still very different. For example, looking at the Organisation for Economic Co-operation and Development indicators, more than three quarters of long-term care recipients receive care at home in Japan and in Norway, against only half of them in the United States [12]. Leichsenring and colleagues analysed the variety of viewpoints towards integrated care in Europe in a comparative study [13–15] and proposed a terminology to reveal these differences. Applying this terminology to Japan, it appears that the community-based integrated care has expanded long-term care insurance services focusing on ‘Case and care management’ and ‘Quality management/assurance’ and is about to grow on a community level through ‘Multiprofessional needs assessment and joint planning’ as well as through ‘Co-ordinating care conference’. The community-based integrated care in Japan is an example of care system built on formerly two independent concepts: the concept of community-based care and the concept of integrated care [16].

In recent years, the idea of bringing together these two concepts has been actively debated in many countries, but only few of them have made real attempts to implement such a system. The Netherlands is one of them, but the success of this system is still a subject of discussion as it is sometimes considered as a Babel tower or as a system between myth and reality [17] even though some successful attempts are reported [18].

The community-based integrated care in Japan is a care system that combines medical and long-term care with approaches similar to other integrated care systems around the world. In other Asian countries, where the ageing of the population is even more sudden than Japan, it is also expected that integrated care system will soon be needed.

These countries, preparing for future ageing trends, are in the process of expanding their care services via the establishment of a Long-Term Care Insurance System. However, if these countries follow a path similar to Japan, the implementation of some sort of community-based integrated care system is likely to become the next critical step to ensure the sustainability of the system.

## Addressing challenges after the reforms of 2006

### Establishment of 24-hour routine home-visit services and need-based care services

Considering that, even seven years after the beginning of the community-based integrated care system in 2006, the system can hardly be considered as fully implemented, the committee decided to provide more concrete recommendations. For example, the community-based integrated care system was described as: ‘A system where long-term care and health care services are provided around-the-clock, 365 days a year, to residents of a community regardless of their type of housing. The services should be chosen by the user and it should be possible to access them within 30 minutes to ensure users with good health, peace of mind and safety in their daily life’.

In this system, a crucial type of service is the 24-hour routine home-visit service and the need-based care services, provided by a team made of nurses and long-term care providers. This team is responsible for conducting home visits in a given community.

The Long-term Care Plan focuses on these new types of services. Since 2006, these services are categorized as a part of community-based services. They are provided to residents by private organisations in charge of a delimited area designated by the municipalities.

24-hour routine home-visit services for nursing care and long-term care can either be provided by the same provider or through the collaboration of two different providers. Providing those services requires a 24-hour call centre and operators but this can be arranged by another provider.

## Promoting collaboration between medical care and long-term care

Concerning residential care, emphasis was put on staff members working in facilities that could provide rehabilitation services and thus contribute to a smoother transition between hospital and home. The Japanese government created incentives through various adjustments of the medical and long-term care systems to increase the collaboration between these two fields. The goal of these incentives is to promote hospital discharge. Concretely, additional remuneration is given to hospitals and long-term care facilities when a patient uses a ‘clinical pathway’ and when information is transmitted during the hospitalisation.

In Japan, discharge management is not clearly established which means that, in most cases, discharge occurs at the initiative of the medical facility. Until now, the continuum of care was sometime interrupted because the Medical Insurance covers the expenditures of medical facilities and the Long-term Care Insurance covers the expenditures of home care and long-term institutional care provider. To solve this issue, financial incentives were created through the Long-term Care Insurance and the Medical Insurance to promote information sharing between hospitals and care facilities and to encourage care providers to actively look for information. Furthermore, a clinical path was also implemented through incentives to promote cooperation between acute care and chronic care within the community. Finally, municipalities were also encouraged to hold their own care conferences on discharge management.

## Coordination between a wide range of care providers

Another crucial part in the system proposed by the committee is to enable users to keep living in their community. In this regard, the committee recommended the establishment of community-based integrated care centres and support networks involving public organisation, Non-Profit Organisation, residents of the community, service providers and community associations working together to enable a prompt provision of various types of services. However, it seems that government institutions are currently too weak to put these recommendations into practice.

This weakness comes from the fact that, at the time of the revision of the Long-term Care Insurance and the Medical Insurance in 2006, many issues were pointed out by research, such as functional deficiencies of community-based integrated care centres.

One cause of these functional deficiencies is that, as it was the case with the UK's reform of 1990, staff in community-based integrated care centres does not have the authority to purchase services for users. The implementation of the Long-Term Care Insurance System did not change the fact that this authority rests with the user, which means that staff are limited to care management consultation and to the coordination of informal services. Thus the relationship with service users remains superficial, hindering the centres’ capacity to fulfil their role of care providers for the community.

Moreover, although community-based integrated care centres are expected to be the core of community care, many of them are not managed directly by municipalities but rather by private sector businesses or Non-Profit Organisations designated by the municipality. It has been pointed out that these centres have less authority to manage services and that they sometime lack crucial information possessed by municipalities.

## Future direction

According to the latest report from the Research Committee on community-based integrated care (2012), the community-based integrated care system is still far from being fully implemented.

For example, a national project was conducted in 2011 to implement 24-hour routine home-visit services and efforts to implement them did increase in 2012 but their implementation level is still well below the expectations of local governments.

The community-based integrated care system distinguishes and reinforces the role of both medical care services and long-term care services. This care delivery system shares many similarities with the definition of integrated care [19] given by the World Health Organization.

One goal of the revision of 2012 was to clarify the responsibilities of municipalities and to promote the cooperation between the medical and social care field through the establishment of mandatory multidisciplinary conferences.

Concerning the collaboration between these two fields, the latest report from the committee gave specific recommendations for service providers. One of the priorities established in this report is to make the delivery of services more efficient and effective.

The focus should no longer be on developing new services, but on delivering integrated services, securing human resources through frameworks such as ‘career up’ and generating an economy of scales concerning human affairs, employment, education and management by promoting business alliances between providers. Providing various services through networked entities established by business alliance or cooperation between various legal bodies is a goal shared with the community-based integrated care system.

Another priority established in the report is the efficient use of institutional resources within the community. In the community-based integrated care system, it is important that long-term care facilities covered by Long-Term Care Insurance System provide not only residential care services but also reinforce the support function of communities.

In the future, these adjustments as well as various reforms should focus on more efficiency. The community-based integrated system is based on an approach similar to integrated care systems seen in other countries, but Japan is still facing issues related to the training of human resources and to the gap between municipalities’ social resources.

## Conclusions and policy recommendations

The community-based integrated care system was implemented to tackle issues that exist also in many other developed countries: the increasing costs of social security and the gap between medical and social care.

In response to an ageing society, the Japanese government has promoted, since 2006, a community-based integrated care system. As explained throughout this paper, the community-based integrated care system of Japan attempts to integrate acute medical care and long-term care. However, from a broader perspective on social services, it is still not clear how to increase the collaboration between social care providers and health care providers. Considering the rapid ageing of the Japanese population, the community-based integrated care system rapidly became a centre of attention but one of the major obstacles to its implementation was the lack of coordination between various providers and the lack of clarity concerning the assignment of responsibilities.

Japan has secured access to health care through a health care system and to long-term care through a separate system implemented in 2000. In 2011, this system had 5 millions 30 thousands recipients (compared to 2 millions 800 thousands in 2000). However, the sustainability of the system remains an issue.

Even though health care services play an important role, Long-Term Care Insurance System also relies on the contribution from families and on the strong Eastern Asian belief that family should take care of each other.

However, along with societal changes such as the increase in unmarried people and single-person households or parent–child separated households, the number of elderly persons living alone has increased.

This explains Japan's attempts to build a community-based integrated care system that supports the delivery of both family care and community care through the coordination of Non-Profit Organisations, volunteer's organisations and private businesses in the community. This system carries great expectations, as one major challenge faced by Japan is the sustainability of the funding system.

The implementation of the system will be adapted to each municipality and will mobilise social resources already existing in each community. However, this implementation process needs to be validated somehow. Further research should focus on finding a way to evaluate the community-based integrated care system and on finding a management strategy to enable the creation of an integrated care system in the community.

## Figures and Tables

**Table 1.**
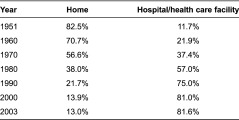
Evolution in terms of places where life ends, 1951–2003

**Table 2.**
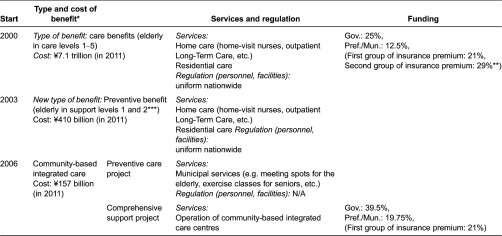
Evolution of benefits and project in LTCIS

Abbreviation: LTCIS, Long-Term Care Insurance System.

*The municipalities are responsible for managing all these benefits.

**The first group of insurance premium concerns people aged 65+. The premium's amount varies depending on the municipality. The second group of insurance premium concerns people aged 40–65 years old. The premium's amount for this group is unique across the whole country.

***Preventive care benefits are provided to elderly persons requiring support, which means people in ‘support level 1’ and ‘support level 2’ categories of the LTCIS (the two lowest need categories). These two categories do not grant access to services in long-term care facilities but grant access to the same types of home care services that people with higher care needs may use (people in care levels 1–5). The category determines the maximum amount of services that is covered by the LTCIS.

**Table 3.**
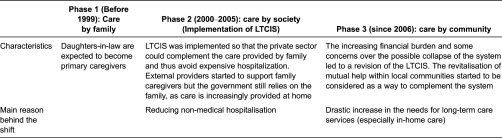
Evolution of care for the elderly and reasons behind the shifts

Abbreviation: LTCIS, Long-Term Care Insurance System.
